# Letter to the editor

**DOI:** 10.1186/1749-8090-5-93

**Published:** 2010-10-29

**Authors:** Luca Bertolaccini, Giovanna Rizzardi, Alberto Terzi

**Affiliations:** 1 Thoracic Surgery Unit, S. Croce e Carle Hospital, Cuneo, Italy

## Abstract

A response to Dango S, Lin R, Hennings E, Passlick B. **Initial experience with a synthetic sealant PleuraSeal**™ **after pulmonary resections: a prospective study with retrospective case matched controls**. *Journal of Cardiothoracic Surgery *2010, **5**:50.

## 

We read with interest the manuscript by Dango et al [1] about their initial experience with a synthetic sealant (Pleuraseal™, Covidien, Mansfield MA, U.S.A.) after pulmonary resections.

Based on our data [2], we agree that this sealant is safe and easy to use and has a significant impact on intra-operative and post-operative air leakage (AL) prevention. Regarding infective complications after use of this sealant, we have not observed any specific complication. In particular, we evaluate the safety and efficacy of Pleuraseal™ for the treatment of parenchymal AL occurring after pleural decortications for empyema thoracis and we do not found increase in the postoperative levels of infectious indexes (Leukocyte and C Reactive Protein). Therefore, this sealant is suitable for routinely use, even in procedures with contaminated pleura.

However, we suggest the use of a digital device for measurement AL instead of the currently used systems to evaluate more accurately and reproducibly AL. This leads to quicker chest tube management decisions because the average size of an AL during the last several hours can be determined. Continuous digital measurement of AL reduces degree of variability of AL score, give more assurance for tube removal, report AL without worry of observer error.

## Competing interests

The authors declare that they have no competing interests.

## Authors' contributions

All authors read and approved the final manuscript.
